# The Influence of Personal Health Data on the Health Coaching Process

**DOI:** 10.3389/fdata.2022.678061

**Published:** 2022-06-14

**Authors:** Heleen Rutjes, Martijn C. Willemsen, Milou A. Feijt, Wijnand A. IJsselsteijn

**Affiliations:** Human-Technology Interaction Group, Eindhoven University of Technology, Eindhoven, Netherlands

**Keywords:** health coaching, wearables, health data, collaborative reflection, coach-client relation, coach-client communication, user study, personal informatics

## Abstract

Tracking health data, for example, through wearable devices or health apps, is increasingly commonplace. Consequently, health coaches (e.g., personal trainers, dieticians) are facing growing numbers of clients who bring their data to the clinic. These data potentially add value to the coaching process, for example, by showing more objective and specific information on clients' behaviors. However, in practice, it turns out to be hard to effectively utilize health data in a coaching setting, and it is not yet fully understood how data affect the coaching process and the coach-client communication. We organized a workshop (12 coaches, 3 clients) and a field study (5 coaches, 6 clients), where we observed coach-client interactions enriched with data. By including both familiar and unfamiliar coach-client pairs, as well as alternating the timing of the data presented (i.e., at the beginning, or halfway through the session), we acquired a variety of data-driven coaching interactions and analyzed this using a mixture of qualitative and quantitative methods. Our results show that data are not “plug-and-play.” There is an extensive process of interpreting and contextualizing data, in which the client has a key role, which is essential to gain relevant and actionable insights from the data useful to the coaching process. We also observed that data affect the coach-client communication on both content and relationship levels. We will reflect on these insights in terms of design recommendations for wearable tracking devices and e-health technology to effectively support health coaches and their interactions with their clients.

## Introduction

Tracking health-related behaviors are becoming increasingly commonplace through the ubiquitous availability of consumer tracking technology, including wearable devices and smartphone applications. Such technology enables a wide range of measurements, from simple step counts to more advanced measures, such as sleep stages and heart rate measurements. Visualizations of these data provide users with a level of insight in, and potentially control over, their own health. These devices are typically presented as e-coaches, thus helping users to achieve their goals rather than merely presenting information. This may include sending motivational messages, recommendations, and comparing the behavior with health standards (e.g., 10-k steps a day) or user-set goals. Thus, when facing health issues or setting health goals, it is assumed that tracking one's data and interacting with e-coaches provide a helpful solution.

The increasing use of tracking technology is inevitably transforming health coaching and the practices of health coaches, such as personal trainers or dieticians. When people visit a health coach, bringing one's self-tracked data will be increasingly commonplace. When people have tracked their health data before meeting a coach, health coaches are facing clients who are potentially better informed and more engaged with their health. Also, the data provide coaches an additional source of information that is essentially different from traditional self-report. As wearable devices are carried along in the daily life of the client, they can provide continuous, high frequency, and *in-situ* measurements, enabling a detailed overview of trends over time (Sqalli and Al-Thani, 2020) contextualized in the daily life of the client (Figueiredo and Chen, 2020). In addition, the nature of the data is different; it is initiated by the client himself or herself rather than suggested by a coach or a doctor, potentially better reflecting the client's perspective and needs. While these benefits are clear, data may not necessarily be informative to coaches or beneficial to the coaching process—it may as well be regarded as a distraction or be perceived as a threat, challenging a coach's expertise. Thus, coaches may find themselves competing against rather than collaborating with the data. Indeed, the current adoption of patient-generated data in healthcare contexts is low (Demiris et al., 2019). Health coaches have reported issues with data in their coaching practice, including disruption of their relationship with the client (Rutjes et al., 2019). By any means, wearable devices and the data they provide are changing the coaching process, which will affect coaches, clients, and their relation in important ways. In the present article, we seek to understand this effect of data on the health coaching process.

### What Is Health Coaching?

Before discussing the possible effects of data on health coaching, we first considered the process of health coaching itself, independent of technology. A systematic literature review of Wolever et al. (2013) reveals some key elements of health coaching. The authors find that health coaching is a patient-centered approach where a patient's goal is leading. Health coaching involves self-discovery, education or self-monitoring, all within the interpersonal relationship with the coach, who is guiding this process (Wolever et al., 2013). This is in line with general definitions of coaching, beyond health, which also considers coaching as an individualized and tailor-made approach, and based on a collaborative relationship rather than one based on authority (Ives, 2008).

Coaching is applied in many application areas related to health, varying from sports and wellbeing to clinical contexts. In sports, recreational and professional athletes are often supported by coaches (e.g., team coaches, personal trainers) to achieve their best sports performance (Cassidy et al., 2009). The term health coaching, on the other hand, is typically used in more clinically oriented contexts, including lifestyle and behavior change support for people with chronic diseases, obesity, or hypertension (Olsen and Nesbitt, 2010; Sforzo et al., 2018). Wellbeing or wellness coaching, while typically taken together with health coaching (c.f., Wolever et al., 2013; Sforzo et al., 2018), often applies to healthy clients. These clients have no medical condition nor a specific sports goal, yet wish to increase their wellbeing or prevent illness. Sports, health, and wellbeing coaching share a similar focus, including lifestyle, nutrition, and physical activity. In the present article, we focus on clients that are essentially healthy, yet who have health-related goals. We exclude medical conditions, such as chronic diseases, as those might narrow down the use of data toward those conditions. We also focus on coaching situations where it is plausible that a client brings his or her own data to a coach, tracked by a consumer device. Therefore, we exclude elite sports from our scope, as, in these contexts, using data is already a common practice, and there are typically more advanced measuring devices available.

The effectiveness of health coaching is studied extensively and shown to have mixed to positive results on improving health outcomes (Olsen and Nesbitt, 2010; Sforzo et al., 2018). The nature of health coaching itself, however, is less understood. What do coaches typically do, say, and recommend? Which techniques do they apply? How long and frequent do coaches and clients meet, and what is the most effective format? What are typical dynamics of a coach-client conversation? Both the reviews of Olsen and Nesbitt (2010) and Wolever et al. (2013) show that health coaching literature often lacks detailed reporting on these aspects, inhibiting systemic evaluation or elicitation of best practices. Nevertheless, the literature provides some suggestions. For example, Olsen and Nesbitt (2010) findings indicate that goal setting and motivational interviewing (MI) typically result in positive health outcomes. Both methods enhance self-awareness, accountability, and confidence. Furthermore, we know from the clinical domain that effective doctor-patient communication is a key to patient satisfaction and positive health outcomes (Ha et al., 2010). It has been argued that clinical conversations should go beyond biomedical topics, including the patient's narrative (Murphy and Franz, 2016) and the patient's values (Berry et al., 2017), to provide appropriate care. A good doctor-patient relationship is characterized by emotional connection and partnership (Dill and Gumpert, 2012), and this is likely to be the same in health coaching.

### The Promise of Data for Health Coaching

For clients, it can be beneficial to track one's health data to gain insight in patterns and relations, possibly supplemented with e-coaching that contains personalized recommendations or motivational messages. It is generally recognized that clients' self-tracked health behaviors are also promising to serve as input for health coaches and healthcare professionals in general. First and foremost, self-tracked data potentially provide coaches with a more objective and reliable view on the client's behavior, compared to the more traditional information source, a client's self-report (Chung et al., 2015; Schroeder et al., 2017). These devices measure continuously, with high frequency, and are situated in the daily life of the client, thus allowing the collection of detailed information of trends in health (Sqalli and Al-Thani, 2020). This may lead to new or deeper insights into the client, and can facilitate personalized care (Figueiredo and Chen, 2020), tailored to a client's specific needs and experiences (Rutjes et al., 2019; Sqalli and Al-Thani, 2020). Combining data of a large group of users allows for novel detection of health issues, which, in turn, can improve the algorithms in health coaching programs, (c.f., Turakhia et al., 2019). So, besides the frequently mentioned benefits self-tracking has for clients, (c.f., Epstein et al., 2020), there are substantial benefits for health coaches and their practices too.

Data may also serve as a memory aid for clients (Figueiredo and Chen, 2020). It is notoriously hard to accurately recall day-to-day behaviors and experiences from memory (c.f., Kahneman and Riis, 2012). When clients report how they have been over the last days or weeks, they draw from their memory, which most likely gives coaches a biased representation. To provide accurate support, coaches need to understand actual behavior and experience, including a client's actual food intake, trainings, if they have been struggling, and how intense that felt in the moment. Bringing data to the coaching session may improve a client's memory retrieval, similar to Kahneman et al. (2004) “day reconstruction method,” where the reconstruction of a day through episodes has been proved to enhance reliable self-report.

These benefits, while important, only consider the individual gains for coaches and clients separately. Basically, coaches have more information; clients are more engaged. Yet, when considering the coach-client relationship and interactions, new benefits emerge. It has been argued that data enhance effective communication between a coach and a client. Mentis et al. (2017) performed a study in which they observed how patients and their caregivers discussed step-count data of the patient. They find that this process of co-interpretation, for example, making sense of outliers and trends in a conversation, supports the re-construction of the patient's narrative. It shows how data serve as an opportunity for clients to share their lived experiences. Also, Rutjes et al. (2019) show that there, potentially, is a synergy between collaboratively reflecting on behavioral data and sharing lived experiences. They argue that, particularly, the ambiguity of behavioral data, i.e., a high step count can reflect intentionally healthy behavior or a broken car, provides relevant cues for meaningful coaching conversations. Figueiredo et al. (2020) interviewed both healthcare providers and patients on their use of data in managing fertility issues and revealed that their data practices are essentially different. For patients, these are mainly driven by emotions, whereas, for providers, this is a mainly rational process. The authors argue that, to effectively utilize data, these different perspectives should be bridged, as both serve different purposes and add unique value. This is in line with several other studies (e.g., Raj et al., 2017; Chung et al., 2019; Pichon et al., 2020), showing that both clients and healthcare professionals bring in their own expertise, resulting in complementary views on the data. Specifically, clients draw from their own lived experiences when reflecting on data, whereas healthcare professionals mostly rely on their medical expertise. In addition, Chung et al. (2019) found that pre-visit notes by clients, based on their food diary data, guided explicit discussion on participants' goals, and thus increased alignment. To conclude, the literature suggests that, in addition to the data themselves, it is the collaborative reflection on the data that adds value, and these collaborative reflections facilitate the alignment of goals, expectations, and perceptions on illness experiences.

### Barriers to Effective Use of Data in Health Coaching

Despite these potential benefits of using data in health coaching, and the growing number of people that engage in self-tracking, the adoption of data in professional contexts is low. Demiris et al. (2019) argue that the adoption of self-tracking tools in clinical practice is still in an “early adopter” stage. The literature points to several barriers that could explain this slow uptake, both from a technical perspective, as well as from the healthcare professionals' points of view.

From a technical perspective, several challenges are identified that may inhibit leveraging these benefits. For example, measurements may be inaccurate (West et al., 2017; Mahajan et al., 2020; Sqalli and Al-Thani, 2020), and tracking devices and their underlying algorithms operate without expert guidance (Mahajan et al., 2020). Furthermore, these data typically comprise clients' health indicators but lack contextual information that is needed to effectively serve as input for personalized health-coaching programs (Sqalli and Al-Thani, 2020).

Health professionals have also expressed a range of perceived barriers, withholding them to use data in their practice. This includes time constraints (Devaraj et al., 2014; Chung et al., 2015; Gagnon et al., 2016; West et al., 2018), privacy, and security concerns (Gagnon et al., 2016; Watt et al., 2019), patients having unrealistic expectations about health professionals reviewing their data (Chung et al., 2015), patients misreading or over-monitoring their data, which reinforces worries (Watt et al., 2019), a lack of expertise to analyze the data (Chung et al., 2015), a lack of familiarity with the technology (Gagnon et al., 2016), and data being incomplete, unreliable or irrelevant (West et al., 2018).

Besides these most practically-oriented barriers, it becomes particularly clear that health professionals want to secure a good relationship with the client when introducing data. They want to avoid data disrupting their contact with their clients (Gagnon et al., 2016; Rutjes et al., 2019). For example, they fear that looking at a screen is misinterpreted as indifference for the client (Gagnon et al., 2016), and they want to prevent an overemphasis on numerical information distracting from the client's subjective experience (Rutjes et al., 2019). This resonates with other researchers' critical perspectives on reducing health to numbers (Van Dijk et al., 2015; Lupton, 2016). When collaborating with hybrid eHealth technology, health professionals stress the need to establish and maintain an empathic relationship with their clients (Brandt et al., 2018). And interview studies with healthcare professionals and patients suggest that, if collaborative reflection on data is not effectively supported, this may reinforce misunderstandings and unaligned expectations (Chung et al., 2016; Raj et al., 2017; Figueiredo et al., 2020; Pichon et al., 2020).

In sum, a large share of the expected benefits and barriers of data on coaching typically goes beyond coaches and clients individually; it is situated within the coach-client relationship. To increase our understanding of the potential effect of data on relational aspects, we discuss this through the lens of the theories of distributed cognition (Hutchins, 2000) and communication theory (Watzlawick et al., 1967).

### Through the Lens of Cognitive and Communication Theories

We should note that the required knowledge for effective health coaching is distributed across the coach, the client, and, possibly, the data from a tracker. This implies that, for gaining a complete understanding that is needed to effectively coach, this distributed knowledge should be shared and coordinated. This process is well-described in the distributed cognition paradigm (Hollan et al., 2000). Drawing from their observations in aviation (c.f., Hutchins and Klausen, 1996), Hollan et al. (2000) argue that cognition needs a larger unit of analysis than just one individual; cognition is distributed across people and technological artifacts. They show how information is transmitted and transformed in such a sociotechnical system, and they argue that cognition is shaped by cultural expectations and social organization. Coaches and clients, like pilots in a cockpit, have expectations of each other in terms of what the other knows and how they are supposed to act based on the information at hand. Coaches and clients even have less established routines than pilots, who can rely on shared training and fixed procedures. This makes mutual coordination between coaches and clients, particularly important to avoid misunderstandings. In this process, coach-client communication is a form of sharing knowledge representations and interpretations of the data on the one hand, and the client's situation and the expected outcome of coaching interventions on the other. This process includes contextualization of data, making predictions, and checking assumptions.

Furthermore, we have seen that in coach-client communication not only the subject matter of what is being discussed is important. It is a key to also consider effective communication in terms of *how* things are discussed, and to situate what is being said within the relationship between the coach and the client. This insight resonates with the communication theory of Watzlawick et al. (1967), arguing that information transmission is always contextualized within a relationship between the sender and the receiver. Every instance of communication can be understood on a content level, i.e., the information that a message contains, as well as on a relationship level. The relationship level of communication comprises, among other things, the sender's expectations on how the message should be understood and what the recipient is expected to do with the information. It basically reveals how the communicators view one another. We have seen that, indeed, effective health coaching is strongly determined by the quality of the coach-client relation, in terms of mutual trust, respect, and investment. Thus, when a client brings his or her data to a coach, it might not only be the data *per se* that inform the coach—it may also be the act of initiating the tracking, the fact that he or she is willing to share, and the way he or she talks about the data, that is informative to the coach. This may signal needs and levels of motivation, dedication, or self-confidence, as well as the need for acknowledgment, expectations of the coaching, or trust in the coach.

### Contribution and Research Questions

Prior literature gives insight into how self-tracking data potentially influence the health coaching process. The current study aims to expand current understandings in two important ways. First, we add to prior literature by exploring the value of data across various conditions. That is, we add data both in the beginning and halfway through the coaching sessions, and we let coaches assess the data both in presence and in absence of the client. This setup allows for understanding the value of data and a conversation, individually and collectively, and comparing those in both qualitative and quantitative ways. It also allows for contrasting coaching sessions that started with data, or started with a conversation, and compare the results when either one is taken as a point of departure. This approach adds to prior work that typically draws insights from sessions where data were available from the start (e.g., Mentis et al., 2017; Raj et al., 2017), resembling our “end-situation” where data and a client conversation come together. In some other studies (such as Figueiredo et al., 2020; Pichon et al., 2020), clients and healthcare professionals are only interviewed individually on their needs and experiences. There have also been studies that observed patients' and healthcare professionals' individual and collaborative interactions with data (Schroeder et al., 2017; Chung et al., 2019). Yet, the current study adds to these studies by an explicit comparison of the coaching sessions with data only, conversation only, and data and client conversation together.

We want to particularly highlight the value of the condition where coaches assess the client's data in absence of the client, as we expect this may yield interesting results. In this condition, we ask the coach to formulate a piece of advice based on the data solely, not knowing the client other than reading his or her goal or question. Essentially, this mimics an e-coaching situation through a Wizard of Oz-like approach, where a coach, be it a human or an artificial one, generates advice merely based on a client's data and goals. Following this up with a conversation with the client, after which the coaching advice is updated, gives insight into not only the value of data but also their possible limitations. It allows us to identify the additional information that a client conversation yields, and it sheds light on the feasibility of e-coaching and specific design considerations for such applications.

Second, we contribute to prior literature with a focus on, essentially, healthy clients who wish to improve their wellbeing and fitness or prevent illness. Prior work on the effects of data on health coaches has mostly focused on medical contexts, for example, working with chronically ill people with irritable bowel syndrome (IBS; Schroeder et al., 2017; Chung et al., 2019), diabetes (Raj et al., 2017), or Parkinson's disease (Mentis et al., 2017). The study of Chung et al. (2019) used both healthy participants as well as chronically ill participants (IBS patients), and they found that the use of data differed across these cases. For the patients with IBS, the focus was mostly on identifying and managing symptom triggers, whereas, for healthy participants, there was more time spent on discussing potential goals and possibilities (Chung et al., 2019). In the current study, we aim to explore how data affect the health-coaching process for healthy clients, where goals are more open-ended compared to health coaching in medical contexts.

We have described how data are likely to have effect beyond merely bringing in additional information, as they also influence relational aspects of coaching. Therefore, we aim to address the effect of data in terms of content and relation separately. Furthermore, while we acknowledge the client's perspective, we will have a main focus on the coach's perspective in our analysis. We seek to broaden the understanding of collaborative use of data, and we believe that this benefits in the first place from exploring how data challenge the roles and working practices of health coaches and how data meet their information needs. Of course, at the same time, we will also address clients' perspectives and needs, and study how data affect the collaborative process as a whole. Accordingly, we aim to answer the main research question:

How do data change the health coaching process?

by answering the following sub-questions:

a. How do coaches and clients relate to the data, i.e., how do they interpret them and utilize them in a coaching session?
b. Can a client's data already be informative to coaches in the absence of explanation or contextualization from the client?
c. How do data change the coaching at the level of coaching content, i.e., topics that are discussed, insights that are gained, and advice that is given?
d. How do data change the coaching at the relationship level, i.e., the roles of the coach and the client in the coaching process, and their relation?

## Materials and Methods

### Study Design

We first organized a workshop to pilot test our setting and measurements, followed by a field study. In both the workshop and the field study, we let coaches interact with clients and their data in various ways. That is, some started with a conversation with the client (*conversation-first* condition), after which the client's data were introduced and discussed. Other coaches started with assessing the client's data (*data-first* condition) in absence of the client, after which the client came in and a conversation started. We both worked with coach-client pairs familiar to each other to understand the effect of adding data into an existing coaching relationship, as well as coach-client pairs unfamiliar to each other. The latter was representative for a coaching “intake” situation and allowed us to study the value of data in isolation, with no background information of the client. Altogether, this resulted in a broad range of setups, covering several phases of the coaching (i.e., intake sessions or further progressed), enabling us to compare and contrast across more data-driven and more conversation-driven sessions.

The design of the workshop and the field study were slightly different. As the workshop served as a pilot test, we made some minor changes in the coach questionnaire used in the field study (indicated with an ^*^ in the Supplementary Material, further discussed in Section Measurements and Data Analysis). The workshop, furthermore, showed that the setting worked generally well, working with the client's data, and integrating this in a conversation with the client was feasible for coaches. Therefore, we proceeded with this design in the field study, with two main differences. First, in the workshop multiple (i.e., 2–3) coaches interacted with one client, in a group conversation, whereas the coaching sessions in the field study were one-on-one interactions between coaches and clients. Second, the coaches in the workshop took part in only one condition (i.e., *data-first* or *conversation-first*), whereas, in the field study, all coaches experienced both conditions. At the same time, the coaches in the workshop were still able to compare across the conditions in a final plenary discussion. Across both studies, we observed the largely similar dynamics and conversation topics, so, for the final analysis, we included the data (transcripts and questionnaires) of both the workshop and the field study.

In this section, we will describe our approach in more detail. This study was approved by the internal ethical committee of our department.

### Participants and Study Procedure

#### Workshop

**Figure 1:**
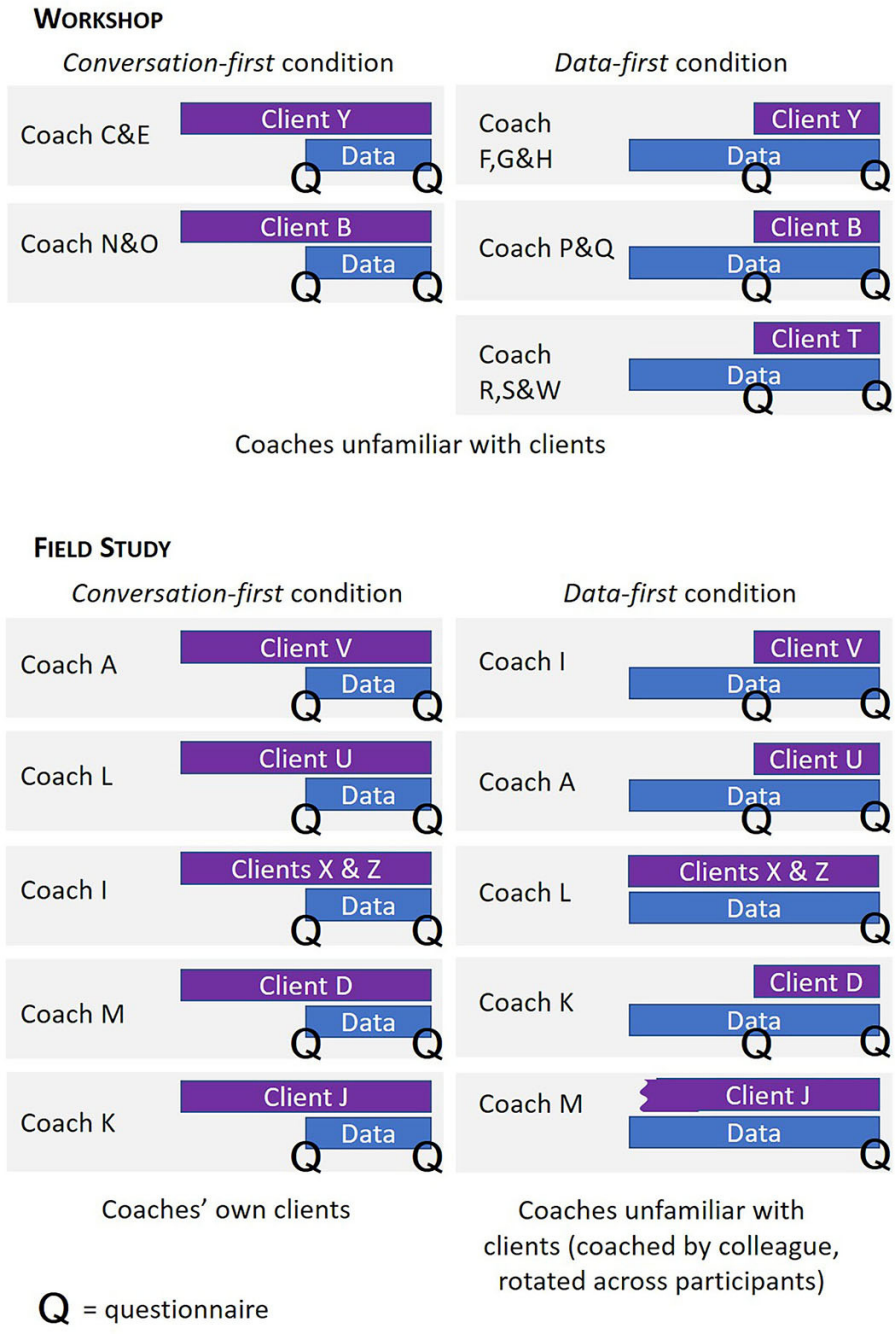
An overview of coaching sessions in the workshop and field study, split by conversation-first, and data-first conditions.

The workshop was organized as one of the parallel sessions at a symposium organized by the sports coaching academy at an applied university, organized for teachers and practitioners in sports coaching. In total, twelve coaches joined with various backgrounds and professions, such as teachers at the sports coaching academy, sports-related community workers, and physiotherapists. There were two workshop rounds: four coaches participated in the first round, and eight coaches participated in the second round. The coaches were split up in small groups of two to three participants and assigned to one client (see Figure 1). The duration of each workshop round was 45 min and was guided by two researchers.

We recruited three clients from our personal network who had a health-related issue or question, although not indicating severe health issues. We invited them to join the sessions as if they were clients visiting a coach and asked them to bring any relevant self-tracked data of any type. All the clients wrote down their questions as input for the sessions. See Table 1 for an overview of their questions and the data they brought. Note that all the coaches were unfamiliar to the clients, as we brought in “stand-in clients” ourselves. All the participants in the workshop, including the coaches and the clients, participated on a voluntary basis.

**Table 1:** An overview of participating clients' goals, questions, and data sources.

	**Client**	**Own coach**	**Coaching goal or question to coach**	**Data source**
Workshop	Y	*n.a*.	I have really low energy after lunch and dinner. How can I overcome that?	iPhone Health app
	B	*n.a*.	I lost substantial weight over the last year, and now I want to maintain my current weight, while still building some strength. What is be a suitable food intake for me?	MyFitnessPal (food intake), Fitbit (physical activity, weight)
	T	*n.a*.	In periods when I do less sports, I lose weight relatively fast. What can I do to avoid this?	Google Fit app
Field study	V	A	Fix knee problems to be able to play basketball again. Lose some weight.	
	X & Z (couple)	I	X: Improve core/body condition/muscle strength, especially after suffering from a discal hernia (lower back). Z: Lose weight, tips to get fitter.	Samsung Gear fit 2 Pro (provided by the study)
	U	L	Lose weight and get toned to look good in wedding dress. Tips to control hunger pangs	
	D	M	Lose weight, have a healthy BMI	
	J	K	Lose weight and live without medicine for high cholesterol and diabetes. Tips for a healthy lifestyle	

#### Field Study

For the field study, we recruited five coaches, of which three personal trainers in a university sports center (A, L, and I), and two dieticians (M and K). Their experience as a coach ranged from 4 to 20 years (*median* = 5), and their average age was 31 years (*SD* = 5.7). Aiming for a realistic setting, we asked the coaches to join the study with one of their own clients that they were currently coaching. This guaranteed that we included coach-client pairs where tracking data were relevant, at least from the coach's perspective. The resulting coach-client pairs had been working together for minimum a month up to a year, with a frequency varying from one time a week to one time a month. None of the pairs had been using self-tracking data before in their coaching sessions, although one of the coaches has recently started exploring the use of self-trackers with other clients. We provided the clients with a health watch to track their behavior, i.e., the Samsung Gear Fit 2 Pro, which they used for ~2 weeks. Again, aiming for a realistic situation, we let clients themselves decide how they would use the tracker. It would automatically track step count, floors, calories burned, and physical activity level, but they were free to take the watch off at any time. Additionally, the clients could decide to switch on the heart rate measurement, track sleep (i.e., keep the watch on at night), and manually track specific sports trainings, nutrition, water intake, and coffee intake. By letting the coaches choose the clients, and letting the clients choose how to use the tracker, we aimed to create situations where tracking meets realistic needs.

Data may not only be added during existing coaching processes; it may also be brought by clients at the start. To mimic this situation, where data are available during an intake session where coaches and clients are yet unfamiliar with each other, we rotated the participating clients across the participating coaches, see Figure 1. The clients' coaching goals are listed in Table 1. The sessions lasted approximately 30 min and were guided by one or two researchers. We compensated the coaches with a €15 voucher, and the clients with a €5 voucher.

### Conditions

#### Conversation-First Condition

In this condition, we sought to observe how data would affect an ongoing coaching process. We instructed the coaches and clients to first have a regular coaching conversation, serving as a baseline. In the workshop, this would resemble an intake situation, as the coaches and the clients were yet unfamiliar to each other. Those clients brought their written questions or coaching goals as input for the session. In the field study, the coaches and clients had been working together for a while. They typically talked about how they had been since the last session, sometimes while stepping on a scale to measure weight in the meantime, or while walking on a treadmill. The coaches were asked to indicate when they had sufficient information to provide advice, after which they filled in the first questionnaire, including their current advice for the client, and an evaluation of the information gained from the client's self-report. Subsequently, the client's data were introduced, and they continued their conversation, now supplemented with data. At the end of the session, the coaches filled in a questionnaire again, asking for any updates in their advice, and an evaluation of the information gained from the data.

#### Data-First Condition

In the data-first condition, the coaches were presented with the client's data at the start, in absence of the client. This enabled us to study the value of mere data, lacking contextualization in a conversation with the client, which essentially represents pure e-coaching. It also provided a baseline, to which we could compare the added value of a conversation. In these sessions, the coaches were assessing the data through the client's devices, i.e., their phone and/or watch interface, and during the workshop sometimes also through web interfaces. The client's question or coaching goal was always shared on article, in some cases elucidated by the clients before they left the room. The coaches were prompted to think aloud as much as possible, and, when they had questions, the researcher helped them to find their ways through the data. As soon as the coaches reported they gained sufficient information from the data to give advice, they filled in a questionnaire, including their advice to the client and an evaluation of the information gained from the data. After, the client was asked to join the coach, and they were instructed to have a coaching session like they would normally have, although informed by the data. They could ask or discuss anything, with the aim of helping the client on his or her goal or question. At the end of the session, again, they filled in a questionnaire, asking for any updates on their advice, and an evaluation of the information gained from the client's self-report.

We should note that condition (*data-first, conversation-first*) and coach-client familiarity are confounded, that is, the *data-first* condition is only applied to unfamiliar coach-client pairs. To understand the mere value of data to coaches, it was not feasible to apply the *data-first* condition to existing coach-client pairs, as coaches' interpretations would, inevitably, be colored by their background knowledge of the client. This did not limit our findings; our conditions did not serve the purpose of a balanced interventional experiment. Rather, we used our conditions to create a variety of realistic situations that allow us to understand the influence of data on health coaching in a broad sense.

As the study progressed, we found that the assessment of data in absence of the client was experienced as very uncomfortable by both coaches and clients. To avoid unnecessary tension, we loosened some constraints in the execution of the protocol, depending on people's responses in the moment. As a result, we allowed the clients' presence, or even help, in some data-first sessions. As the goal of the conditions was to introduce variance in our data, rather than making a strict comparison across conditions, we could permit these deviations. More specifically, Coach L was assessing the data of Clients X and Z in their presence and with their help, Client D was not sent out of the room when Coach K was assessing here data (which limited the think-aloud, obviously), and Client Jo was enthusiastically explaining her data to Coach M from the beginning, which we deliberately let happen (see Figure 1).

All clients, except Client T, participated in both the *conversation-first* as well as the *data-first* condition, with different coaches. The coaches in the field study also participated in both conditions, with different clients. Four out of five coaches had the *conversation-first* session before the *data-first* session. The coaches in the workshop were assigned to one condition; however, in the last workshop round, we ended with a short (5-min) group discussion to reflect on the differences across the conditions.

### Measurements and Data Analysis

All sessions were audio recorded and transcribed verbatim, allowing for a detailed analysis of the coaching sessions, including the dynamics of the coach-client conversation, their reflections on the data, and the coaches' questions and advice to the client. The transcripts of the sessions were analyzed through the established qualitative research method thematic analysis (Boyatzis, 1998) in the software package MaxQDA. In this process, we used a mostly inductive approach, comparing and contrasting across the four subsamples of coaching sessions, being data only, conversation only, data after conversation, and conversation after data. This supported our goal of understanding the value of data and the value of a client conversation, individually and collectively. We expected that both would have their unique contributions to the coaches and the coaching process. Emerging themes that differentiated these subsamples were iteratively and systemically tested against the corpus of transcripts. Intermediate versions of themes were frequently shared and discussed with the research team (HR, MF, MW, WI) to check their validity and relevance. HR coded all data, and when the final thematic codes were set, MF coded 20% of the data, resulting in an Inter-Rater Reliability (IRR) of 82%. All disagreements were resolved by discussion. Most disagreements were resulting from the different but consecutive codes “understanding behavior” and “understanding experience.” One may argue that these codes could be merged because they are very similar. When doing so, the IRR increased to 88%.

The coaches and the clients filled in two questionnaires, enabling us to systematically compare and contrast the value of data, conversation, and their combination. One halfway through, i.e., right before the data or client was introduced, and one at the end of the session. For our results, we only analyzed the coach questionnaire, as our main focus was on the coach's perspective. In this questionnaire, the coaches were asked to write down their advice (intermediate or final), which we included in the thematic analysis. Furthermore, we measured their perceived usability, objectivity, clarity, and relevance of the information source at hand (i.e., data or conversation), on a 5-point Likert scale, and the extent to which the information was sufficient to provide coaching, also on a 5-point Likert scale. The full questionnaires are provided in the Supplementary Material. For the analysis, multilevel models are applied, with the coach as a grouping variable (four measurements per coach in the field study, two measurements per coach in the workshop). The type of information (data or conversation) and timing of the measurement (halfway or end) were used as predictors. Note that, depending on the condition, the value of data was measured halfway (*data-first* condition) or at the end (*conversation-first* condition) of the session, and *vice versa* for the conversation. This allows us to also test possible interactions, i.e., where data evaluated differently at the end, when the coaches assessed the data within a conversation with the client, compared to halfway, when only assessed in absence of the client?

Our sample is too small to draw reliable conclusions from quantitative analyses solely. We mitigate this problem partly by having repeated measurements, allowing for testing effects within-person. Still, we should interpret the findings from purely the quantitative analysis with caution. However, in combination with the qualitative analysis, where we also contrast and compare the value of data vs. a client conversation, it may add valuable information. More specifically, in the questionnaire, the coaches judged the value of a client's data *relative to* a client conversation, for example, in terms of clarity and objectivity of information. Since these results are of similar kind as the results from the qualitative analysis, be it measured in a more systematic way, we present the results as additional evidence. This should be considered within the bigger picture of the results based on both qualitative and quantitative evidence and insights.

## Results

The coaches and the clients were generally open and cooperative during the sessions. The sessions with familiar coach-client pairs showed seemingly natural coaching conversations, and, in the sessions with unfamiliar coach-client pairs, they seemed motivated to get to know each other. The researchers were generally accepted as observers, although, often at some point, the participants seemed to expect a specific task on what to do with the data, and we reiterated that we expected them to use them freely according to their own needs and interests. This was typically followed by lively discussions, driven by data to a more or lesser extent, which we will reflect on in more detail in the sections below.

The study setting seemed to be natural to the coaches and the clients, with the notable exception of the first phase of the data-first condition, where the coach assessed the data in absence of the client. For the clients, it was awkward to give away their phones—a highly intimate and personal device (illustrated by a client stating with a mixture of being funny and being nervous: “*if there are messages coming in, do not answer them!”*), and the coaches felt put on the spot to assess data and come up with advice without input from the client (“*this is completely against my principles!”*). The moment when the client entered the room again was often accompanied with ice-breaking statements like “*when will I die?”* or “*are you going to analyze me now?*.” Therefore, as the study progressed, we decided to lose this constraint, and allowed the clients to be present or even help while the coaches were assessing their data.

**Figure 2:**
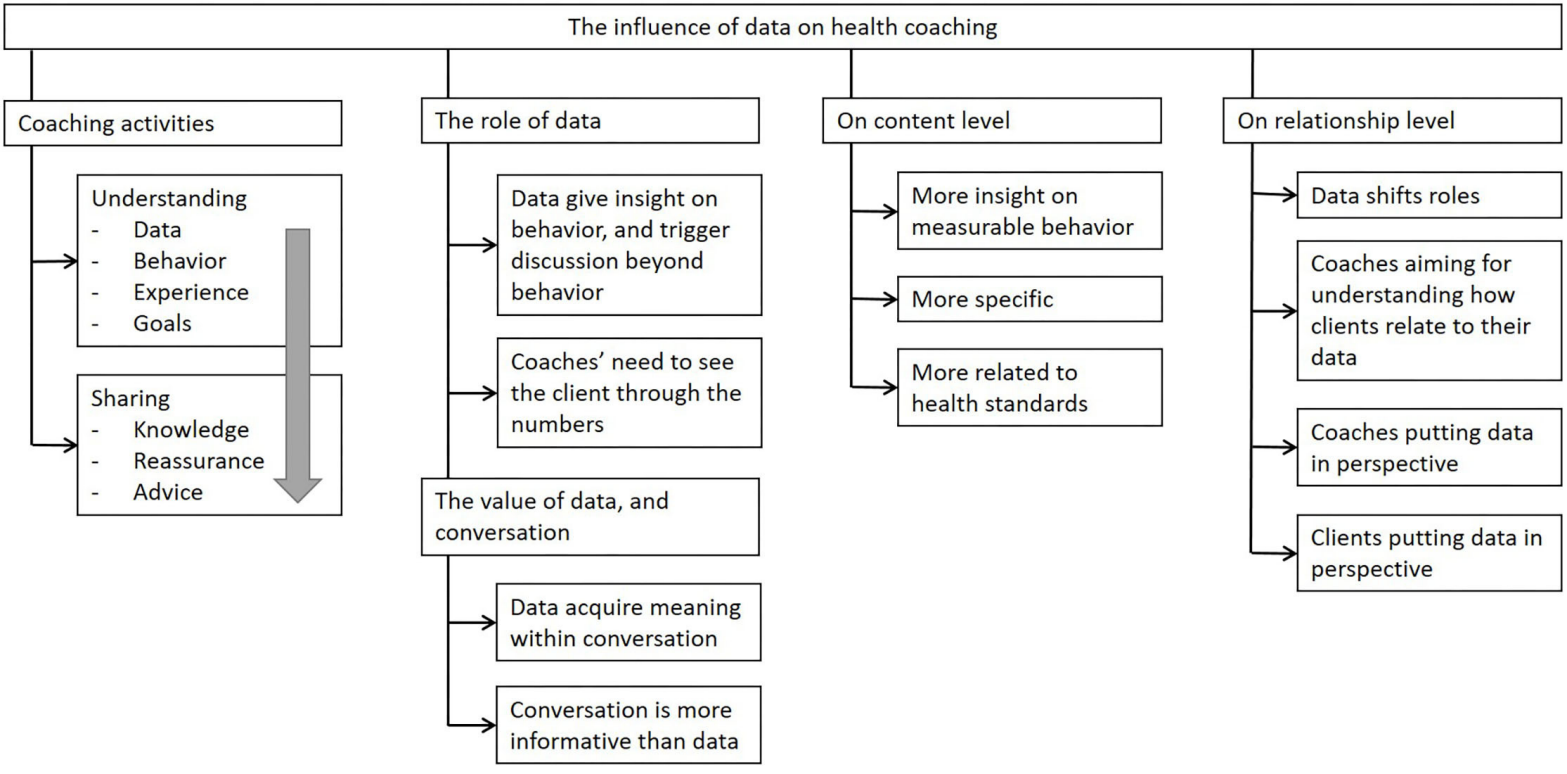
An overview of results in four main categories: (1) incremental coaching activities and (2) the role and value of data therein, (3) how data affect health coaching on the content level and (4) on the relationship level.

Figure 2 provides an overview of the results, both from the thematic analysis and the questionnaires. In the sections below, we will describe the incremental coaching activities in a session and the role and value of data therein, followed by a description on how data are affecting health coaching on content as well as on the relationship level.

### The Incremental Activities in a Coaching Session

To understand the context wherein we introduced the data, we first provide a general description of the coaching sessions in terms of the dynamics and activities.

#### From Understanding to Sharing

Throughout all coaching sessions, we identified two main types of activities. First, there were activities targeted at understanding. Here, the coach mainly asked questions and listened, trying to build up an image of the client's data (if available), her recent behaviors and experiences, current status and goals. As soon as there was a sufficient understanding on these aspects, the coach moved toward activities revolving around sharing. Here, the coach shared her knowledge and expertise, reassured the client and gave her compliments, and gave specific advice. An overview of these activities, including example quotes, can be found in Table 2. Typically, during “sharing activities,” the coaches took a more leading role in the conversation compared to during “understanding activities.” Still, it also happened that they took a more facilitating role, trying to let the clients themselves come up with actionable insights.

**Table 2:** An overview of coaching activities, including example quotes.

	**Coaching activity**	**Example quotes**
Understanding	Data itself and context wherein data were tracked	- For how long did you track? - Did you not move here, or were you just not wearing your watch? - Are these activities (walking, cycling) automatically tracked, or did you manually switch it on? - What does this blue line represent?
	Behavior (what-questions)	- How many times a week do you work out? - What kind of sports do you do? - What did you have for lunch?
	Experience (how- and why-questions) and daily life	- It was on the last week when your steps dropped; can I know why? - Do you wake up fresh? - Do you like to play tennis? - What kind of work do you do?
	Goals and current status	- Are you satisfied with you weight now? - I see that your activity levels are already quite good. Do you have a certain goal with that? - Did you have enough energy this week to do what you wanted to do?
Sharing	Knowledge, expertise	-Your lack of energy can be caused by so many factors, it may be your sugar intake, stress, or screen time. The impact can be different for everyone, so we need to explore what works for you - If you make soup yourself, you could try to make it low in salt by using […]. - Let me explain you how it works with sleep cycles - This heartrate is normal when you do an intense training
	Reassurance and compliments	- I know it's hard, but you did it before, so I'm sure you can do it again. - Very good, the average is 6,000 steps a day, well done! - Don't be too hard on yourself if you did not reach your goal for a day, look at what you've already achieved!
	Advice	- Add some higher intensity activities - It's always good to work out together, other people can motivate you - Try to walk a bit more. For example, at work, use your break to walk around the company - If you see in your food tracker at the end of the day that you have some room left in the calories, first check whether you've had all the required nutrients, and avoid eating “empty calories” like a cookie

#### From Data to Client

**Figure 3:**
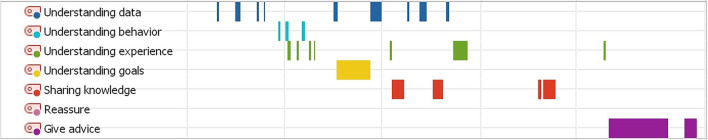
Typical blueprint of a coaching session, illustrating the incremental pattern from data oriented to client oriented, from understanding to giving advice. All codes referring to coaching activities are highlighted; x-axis represents time in the session (A coaching session with Coach L and Clients X and Z, both data and clients present).

The coaching activities showed to be typically incremental, which is illustrated by the code-line visualization of the session of Coach L and Clients X and Y in Figure 3. Activities aiming at understanding occurred mainly at the beginning of a session, and sharing mainly at the end, while it also happened that the coaches were switching back and forth when new knowledge gaps emerged. Furthermore, we found incremental levels of understanding, gradually moving from data or behavior oriented to client oriented (see also Table 2). A typical sequence started with coaches seeking after understanding the data themselves (e.g., this number is your step count?), followed by understanding the clients' behavior (e.g., how often do you walk?) and then soliciting their experience (e.g., do you like to walk?). Finally, they aimed at understanding this information, considering their current status and goals. E.g., does the particular behavior or experience disclose the client's struggles and challenges? Or, can it potentially contribute to the client's goals and wellbeing? Only after the coaches had a sufficient understanding on how the clients were doing in light of their goals and challenges, they were ready to move to sharing activities, ultimately giving advice.

### The Role and Value of Data

In this section, we consider the role of data within the dynamics of a coaching session. We will situate the role of data within the incremental activities of a coaching session, as well as reflect on the value of data, conversation, and their combination.

#### Data Are Considered Beyond the Behavior They Represent

Most notably, data mostly led to insights into the behavioral level. For example, Coach L already concluded in the 3rd minute of the session “*So basically, during the week, you're biking about 45 min to the office. And then, in the weekends, you're really walking a lot more.”* But such a straightforward understanding of the tracked behavior never appeared to be enough for the coaches. They were clearly seeking after the clients' reasons for their behavior. For example, when Coach P noticed the client had an unhealthy snack, she solicited for her reasons*: “was that a moment of weakness, or, perhaps, you did not have a healthy alternative at that moment?”* after which the client reported “*I'm just exploring how to find a sustainable diet*; *I think a snack every now and then should be acceptable within a normal healthy lifestyle*.” This illustrates that the reasons for certain behavior were essential for coaches to accurately interpret it. Interestingly, the coaches sometimes even started discussing seemingly straightforward behavior to increase their understanding of the client. For example, while Coach G already knew the answer from the data, she still asked “*how often do you use the stairs instead of the elevator? And is that only in the morning, when you still have the energy, or do you do it in the afternoon too?*.” Asking the client these questions provided the coaches with extra information on *how* the client answered them, and the client's answer added the context of a specific colleague always motivating her to take the stairs.

Thus, while data mostly added information in terms of the client's behavior, they also prompted conversations on the experience of those behaviors, the context wherein the behavior was performed, the triggers that motivated the client to execute the behavior, and their personal value judgment on the behavior. In this sense, the data provided input and tools for the coaches on all levels of understanding, from low-level behavior to higher-level lived experiences and goals. Yet, data were rarely self-explanatory. Higher-level insights into the client were only gained through effective communication, where interpretations were shared, and data were collaboratively reflected on.

#### Coaches' Need to See the Client Through the Numbers

The coaches showed to be keen on moving their focus from the data to the client as soon as possible. They showed little interest in the numbers *per se*; for example, they rarely engaged in efforts to analyze the data themselves. Rather, the coaches quickly shifted to what the data meant in terms of the client's narrative by soliciting the client's experiences. They easily disregarded data when there was no clear connection with the client's goals and experiences relevant for coaching. For example, in one coaching session, the client's goal of losing weight clearly had a highly emotional connotation, making the step count data not only irrelevant but also very inappropriate to discuss. In this specific session, the data were barely discussed, other than an abstract discussion on how self-tracking at some point could be helpful as a motivation or to get more insight. Concluding, we observed the coaches' urge to move away from the numbers to the client as soon as they reasonably could. In this light, the data did slow down the coaching process in some cases, being something that needed to be clarified before they were able to focus on the client in ways they considered more meaningful.

#### The Individual and Combined Value of Data and Conversation

Next to our qualitative results from the thematic analysis, the results from the questionnaire provide additional insight when contrasting and comparing the value of data vs. conversation. In this section, we present the coaches' perceived usability, objectivity, clarity, and relevance of the information source at hand (i.e., data or conversation), and the extent to which they felt they had sufficient information to give appropriate advice. Multiple measurements per coach over the various conditions allowed us to measure effects within-person, and compare the perceived value of data *relative to* the perceived value of a client conversation, when presented separate or together.

**Figure 4:**
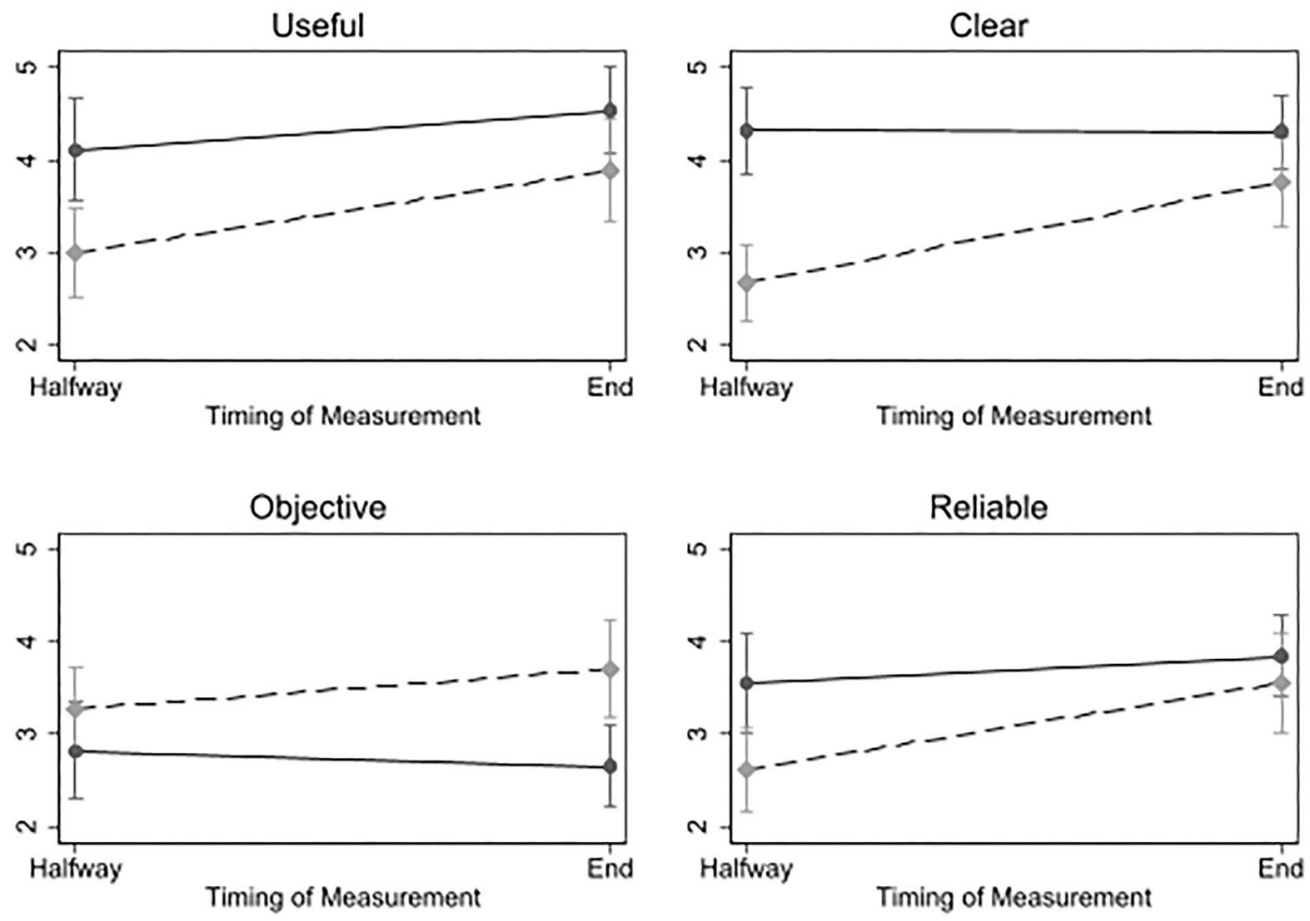
Results of multilevel models, showing coaches' evaluations of data (a dashed line) and conversation (a solid line), halfway and at the end of the coaching sessions.

We first inspected the main effect of conversation vs. data for each of the dependent measures. As Figure 4 shows, coaches value conversation as more useful (*p* = 0.001), clearer (*p* < 0.001), and more reliable (*p* = 0.014) than data, whereas data were valued as more objective (*p* < 0.001). Furthermore, there was one significant interaction effect (*p* = 0.015); data were valued as significantly clearer at the end of the study when assessed within a conversation with the client, compared to halfway, when assessed in absence of the client. Thus, data apparently became clearer when contextualized in a conversation. Similar interaction effects with the other outcome variables were not significant.

Halfway through the session, when the coaches had faced only one source of information (i.e., data or conversation), we measured the extent to which the coaches felt they had sufficient information to give appropriate advice. The coaches scored significantly higher on having enough information after solely a conversation (M = 3.56) compared to after-solely data (M = 1.69, two-sided *t*-test^1^, *p* = 0.001). At the end of the sessions, we measured the extent to which the coaches felt they had more information than in the first half of the session. Both a conversation (M = 4.54) and data (M = 3.78) showed to add more information, and no significant difference was found between them (two-sided *t*-test, *p* = 0.085). So both conversation and data seem to supplement each other, and the conversation was valued as more informative by itself.

In conclusion, the analysis of the questionnaire, while based on a limited sample, shows a coherent message that is largely in line with our qualitative findings. It suggests that coaches generally gain more information from a conversation with the client, compared to assessing the client's data. The results show that data do acquire value when situated within a conversation, allowing for discussing the data and sharing interpretations. Indeed, when only a client's data were available to the coaches, in absence of the client, many coaches expressed difficulty to interpret the data and formulate advice. For example, Coach K reflected: “*I have tons of questions, why this, why that*.” And when the client was introduced after the data assessment, the coached valued the conversation as “*very valuable”* (Coach L) in order to “*get a clear picture of their goals”* (Coach L) and to “*learn the reason of the data results”* (Coach I).

### Data Changing Content-Aspects of Coaching

We have described which role and value data may have in a coaching session. In the next sections, we specifically focus on the influence of the data on the content aspects of the coaching. Specifically, data put forward different topics to be discussed, leading to new insights. Mainly, data were adding insight into measurable behavior, making conversations more specific, and more driven by health standards.

#### Data Provide Insight Into Measurable Behavior

Although we have seen that the mere value of data is limited, our results do reveal the value of data according to the coaches. The responses to the open question regarding the value of data showed data gave coaches “*an overall understanding of the client's activity level”* (Coach A) or “*an indication of their basic health stats, such as rest heart rate and activity levels”* (Coach L). Indeed, pieces of advice based on solely data often included those “basic health stats,” such as “*try to aim for 10,000 steps a day”* (Coach G). Interestingly, when this advice was updated after the client conversation, this sometimes showed to move away from the data, i.e., “*learn to trust your body and rely on your own intuition”* (Coach P), while, in other cases, the focus on data was only strengthened, i.e., “*monitor other things that potentially explain weight, nutrition, and sleep habits, and see if you can find relations there”* (Coach H).

#### Data Provide More Specific Cues for Coaching

While we have previously discussed the coaches' need to see the client through the numbers, at the same time, we observed that, in some cases, the data showed to provide an additional lens on the client, revealing new information relevant to the coaching. When the coaches and their clients were discussing the data, this sometimes resulted in topics which clearly would not have been discussed without the data. These topics were often very specific and highly contextualized in the daily life of the client. For example, when Coach A was checking the food intake of her client, she asked: “*you eat Kung Pao? Did you make it yourself, or…?”* The client replied: “*No, it was from a Chinese restaurant. I was eating out (…)”* Coach: “*How often do you eat out?”* Client: “*It really depends, like, sometimes, it can be once a week, sometimes it can be a month that I don't eat out at all.”* Coach: “*Okay, so maximum once a week. That's okay.”* The topic of cooking yourself or eating out, and the corresponding value judgment that it is okay to eat out as long as it is not more than one time a week would most likely not have emerged without the data. In another example, the data served as memory aid for the client. Coach M asked “*Sunday, uh Monday? What was going on this… You were working? The 20th. 13,222 steps.”* Client: “*Uhm… I have to check what I did that day; I cannot remember. Ah, then I had a day off! I had a funeral, and, in the morning, I made long walk with my neighbor.”* Such specific statements provide the coaches with useful cues to deepen their understanding of the client's daily life, social environment, and lived experiences. While triggered by data, this provides insights beyond data into behavioral and even experiential levels, and a starting point for meaningful coaching.

#### Data Trigger Comparisons With Health Standards

Also, we observed that data triggered conversations on standards and norms; on what is “normal” for a person, or outside of a normal range. Particularly, when the coaches were checking the data without the client, thus lacking the background of the data, they were typically comparing the data with health standards. As Coach F reflected: “*I need to ask many questions first. The only thing I take from the data now is whether she meets the standards for physical activity.”* Also, when the clients were present and collaboratively discussing the data, comparing the data with the standards was a common occurrence. Such standards regarded daily step counts, water intake, light and intense physical activity, sleeping time, and sedentary time.

### Data Changing Relationship-Aspects of Coaching

Data did not only change the content of the coaching; they also affected the relation between the coaches and the clients. Data shifted roles, and both the coaches and the clients were keen on understanding how the other would relate to the data. This informed their own efforts to put the data in the right perspective.

#### Data Shift Roles, Typically Putting the Client More Central

In the *conversation-first* condition, where data were added within an ongoing session, the clients often took a leading role in the conversation as soon as the data were presented. They felt ownership over them, because they have been living and working with the data over the recent weeks; thus, they took their responsibility to explain them to the coach. This was not only driven by the clients. It also happened that the coaches explicitly asked the clients what they wanted to discuss regarding the data, such as Coach K: “*is that the most important for you to evaluate now? The food?”* Not only the data pushed the clients in a more leading role—it also happened that the data themselves were leading in the conversation. That is, some coaches systematically “checked off” the tabs in the menu of the tracker (i.e., “*Let's see, what else you have tracked. Ah, sleep, let's have look”*), following the data rather than their own agenda.

#### Coaches' Efforts to Understand How the Clients Relate to Their Data

The coaches showed to be motivated to understand how the clients perceived their data and felt about their data. They frequently asked the clients how they experienced the tracking, for example, how they used the tracker throughout their day, how often they checked the numbers on their watches or phones, and whether it motivated them or made them nervous. Furthermore, the coaches derived information from observing how the clients engaged in the tracking. For example, one client brought large amounts of data (self-initiated) to the workshop, very detailed and over a long period of time. Based on this, the coaches drew the conclusion that this client was very persistent, and, at the same time, risking, focusing too much on the numbers rather than on how she felt. And, indeed, this became an important topic in the coaching session with the client. Another client forgot to bring her phone to both sessions, which forced the coaches to look at the data on the small interface on the watch. The coaches attributed this to a possible lack of engagement or fear of showing her data. Thus, how the clients related to their data was informative to the coaches.

#### Coaches Putting the Data in Perspective

Mostly, as a response to the clients' worries, the coaches put the data in perspective. They typically reflected on how they understood the data, how the data related to their knowledge, and then giving their value judgment on the clients' behavior. For example, when a client reported “*it shocked me to see that my natrium intake is apparently too high*,” Coach M explained her knowledge on natrium, how it is different from salt, and what it meant in context of the high blood pressure of the client. Then, the coach challenged the threshold for natrium in the app. She recalculated it using her own formula, concluding that there was no reason for the client to worry. In another example, a client expressed “*when I eat out, the next day, my weight increased with 1.5 kg. This cannot be all fat, can it? And I did not even take a desert! This really worries me. How is this possible?”* Coach M guaranteed that this could, indeed, not be only fat. She shared some knowledge on how it could be due to salt intake, but, mostly, the coach was trying to draw the focus away from the weight measurement. She explained that weight may vary a lot on the short term, and that, therefore, it only makes sense to measure it with longer intervals. This pattern frequently happened across the coaching sessions. Data, supplemented with clients' thoughts and feelings on them, provided the coaches the opportunity to reassure the clients, make compliments, or share additional knowledge. In some extreme cases, the coaches even recommended to stop tracking, to put their minds at ease, and to focus on the benefits their healthy lifestyle brings them.

#### Clients Putting the Data in Perspective

Also, the clients showed to be motivated to put their data in the right perspective. First and foremost, the clients frequently reflected on the reliability of the data and tried to guide coaches to interpretations that they found accurate. For example, when checking the number of stairs climbed, a client reflected “*it doesn't recognize this. I have two floors at home, and I go up and down so many times a day, but it only recognizes a few times.”* Or, when Coach M found high-calorie peanut candy in the nutrition list, a client responded surprised “*Oh, that is a mistake; that should be the healthy nut bar! Peanut candy, oh no, no I wouldn't eat that.”* And, when Coach A read out loud that the client drank 6 beers that week, the client responded, “*Now you making it sound like I had a beer every night!”* and explained that it was actually due to a party at work. Thus, the clients showed to be highly invested in making coaches understand and interpret their data accurately and with the right nuance. They cared about their image that the coaches would build from their data.

Lastly, the clients showed to have expectations on how the data would be valued compared to their self-report. The coaches mostly focused on self-report as their main source of information, but, in the rare cases, where they had a stronger focus on the data, this was not always appreciated by the clients. For example, when Coach A said while checking the data: “*You didn't eat much yesterday*,” the client replied rather frustrated: “*That's what I said!”* She seemed offended that the coach did not take her word for it, and that the data apparently added information to her self-report.

## Discussion

Personal tracking data play an increasingly important role in current healthcare practices. Healthcare professionals, sports coaches, and lifestyle coaches are expected to benefit from the additional insights that the availability of data may bring. However, evidence is accruing that the mere insertion of more data into a health-coaching practice does not linearly result in better outcomes, or, indeed, a better process. The focus of the current article is to improve our understanding of the role of data in the health-coaching processes, and how this affects the role of the coach. Specifically, we look at how clients' self-tracked data influence health coaching, both in terms of coaching content and the relationship between coaches and their clients. In a workshop and a field study, we observed coaching sessions where personal health data were added in various ways; at the start and halfway through the session, in the presence and absence of the client whose data were being inspected, and within familiar coach-client relationships or in an intake situation where the coaches and the clients were unfamiliar to each other. Our real-world observations enabled us to situate our insights into the data within the dynamics of the health-coaching process. In addition, we gained insight into the value of data and conversation, individually and collectively, by presenting the coaches with the clients' data and client conversations in various orders.

Throughout the study, the coaching sessions demonstrated a pattern of incremental activities, moving from an initial need for low-level understanding of data and behavior toward understanding higher-level client aspects, such as the context wherein the behavior was performed and how this relates to the client's goals and experiences. Only after the coaches gained sufficient understanding, they gradually moved to sharing knowledge and giving advice. Within this process, the coaches and the clients showed to be in a continuous process of negotiation on the meaning of the data, where they were motivated to put the data in the right perspective for themselves and for the others. For example, the coaches were seeking to connect the data to the client's goals and experiences, and the clients were trying to make sure that the coach would build an accurate and nuanced picture of them based on the data. Furthermore, we observed that the presence of data could also bring up different topics. These topics were typically more specific, more related to health standards, and more oriented to measurable behavior. Yet, the data were rarely self-explanatory. Both our qualitative and quantitative analyses strongly show that collaborative reflection on the data, where interpretations are shared and data are contextualized within the clients' narrative, was required for data to be meaningful and useful in the coaching process.

### Data Are Not “Plug-and-Play”

Wearable tracking devices and e-coaching applications are mostly presented as finished products or solutions. They are built on the premise that the personal tracked data provide an objective view on behavior, as opposed to subjective experience and biased self-report. Through a set of rather linear cause-effect relationships, data are expected to enable detection of trends and correlations, resulting in insight and ultimately, effective coaching. This implies that such data must add value for health coaches as well; after all, more information is assumed to be better. Our results paint a more nuanced picture. While data do bring certain value to the coaching process, this value does not come from the data in and of themselves. Data are not plug-and-play; they need contextualization from the client to be meaningful in the coaching process. Specifically, merely presenting behavior does not reveal, among other things, why the behavior was performed, whether it was a pleasant experience for the client or a struggle, which belief or contextual situation triggered the behavior, and whether the behavior was beneficial at all in terms of the client's goal and narrative. We argue that the inherent value of data is very limited; data do not have value *because* they are objective. Rather, data only acquire meaning when *seen through a subjective perception* of the client, as part of a dynamic and collaborative process of meaning making, involving intrapersonal, interpersonal, and data-driven reflections and interactions.

We expected that the data would serve as memory aid for clients (Figueiredo and Chen, 2020), and, indeed, our results show that the clients recalled specific events and experiences when discussing data. The coaches, however, found it hard to gain actionable insights from the data. Both our qualitative and quantitative results show that data are more informative to coaches when assessed in combination with a client conversation. Building on prior findings (e.g., Mentis et al., 2017; Rutjes et al., 2019; Figueiredo et al., 2020; Pichon et al., 2020) that emphasize the value of collaborative reflection on data, our results show that data provide useful conversation starters and facilitate sharing lived experiences. Our results additionally show that data disconnected from interpersonal exchange typically result in more questions than answers. This effect is likely amplified by the character of health coaching for healthy clients, where the goal and thus the use of data are more open-ended compared to more medical contexts. We discuss this further in Section Difference Between Healthy Clients and Patients.

### E-Health Technology Should Not Merely Focus on Transferring Information

When designing self-tracking devices and e-health technologies, our results show that it is a key to facilitate broader collaboration than merely sharing data. To be able to effectively use and interpret data, we should allow these data to acquire meaning within a coach-client conversation. In this conversation, we have to acknowledge that coaches and clients are not only sharing information; at the same time, they are establishing and maintaining a relationship (c.f., Watzlawick et al., 1967). Data are added to a dynamic interplay between a coach and a client that is subject to trust, expectations, empathy, and investment. This calls for a broader view on self-tracking devices than merely a computational system. Drawing from distributed cognition theory (Hollan et al., 2000), we can consider the coach, client, and tracking device as a sociotechnical system, wherein it is important that all agents are enabled to effectively share and utilize their unique knowledge representations of the data and the status and needs of the client. Thus, these technologies do not provide one-on-one solutions, and data do not provide answers. Rather, these technologies and the data they bring forward are enablers of a good coach-client relationship and effective communication, together, resulting in effective coaching. Health coaching is, after all, based on collaborative rather than hierarchical relations (Wolever et al., 2013).

Thus, data visualizations and dashboards for clients and their coaches will need to support the coaching process and the coach-client relation with giving the right cues. Specifically, our results show that information that is very specific and well-contextualized (e.g., specific food or exercises; where the client was and with whom) yielded useful coaching conversations. Our results also show that such specific information alone is not enough, even seemingly self-evident behavior was still frequently questioned by the coaches and discussing this led to deeper insights into the client. Furthermore, presenting this information is only helpful when it is meaningful in terms of the client's status and goals. For example, when a client's struggles are rather emotional, presenting simple behavior, such as step counts, can turn out to be very inappropriate.

Prior literature typically points to low-resolution, incomplete or unreliable data as main barriers for data to effectively serve as input for health coaching (West et al., 2017; Mahajan et al., 2020; Sqalli and Al-Thani, 2020). Yet, our results extend these findings in that, for data to be useful, it is not only a matter of measuring more consistently and more accurately. It is a matter of measuring those things that are relevant to a client's goals and struggles, and to enable him or her to explain these experiences through the data. This can even go beyond the numerical information that data provide; we have seen that the simple fact that clients bring large amounts of data, or no data at all, can entail important information for coaches. Coaching advice is often based on information at the level of the client's experience rather than his or her data. Thus, data are, mostly, a means to an end, in which collaborative reflection on the data is essential for coaches to understand the data through their clients' eyes, and to provide appropriate support. A coaching process is an inherently social process that goes beyond an optimization problem based on data.

### Difference Between Healthy Clients and Patients

This brings us to reflect on what is unique about health coaching when it comes to the value and use of data, compared to the more frequently studied medical domain. Health data in coaching settings have so far been typically studied with patients with medical issues or (chronic) diseases, such as Parkinson's disease (Mentis et al., 2017; Nunes et al., 2019), irritable bowel syndrome (Schroeder et al., 2017; Chung et al., 2019), endometriosis (Pichon et al., 2020) or fertility issues (Figueiredo et al., 2020). In such cases, it is relatively clear which metrics are relevant to track, and also what counts as “good” or “healthy” on these metrics. For example, in a study on step count data with patients with Parkinson's disease, it is implicitly assumed that more walking is better (c.f., Mentis et al., 2017) and, in various other studies, is assumed that the less symptoms, the better (e.g., c.f., Schroeder et al., 2017). In health coaching with healthy participants—who do have health-related goals yet no disease—this is less clear. For example, the clients in our study were generally aiming for a healthier lifestyle, lose some weight, and become fitter. Underlying these goals, we observed a broad range of issues, varying from self-esteem issues to sports performance goals. This gives the task of health coaching a relatively open-ended nature, in which success is not always clearly defined. Perhaps, for instance, feeling better about oneself is already sufficient, regardless of an actual change in behavior. It is, therefore, not self-evident which metrics are relevant to track, and what appropriate targets are for those metrics.

When comparing our results to other literature, exploring the value of data in the medical domain, we observe some similarities and differences. First, in both settings, thus applying to both healthy clients and ill patients, it is considered a key to contextualize the data in the lived experience of the client to make effective use of the data. Specifically, our findings around the value of a client conversation and the “need to see the client through the numbers” align with findings from the medical domain, specifically using the data to obtain a holistic view on the patient (Pichon et al., 2020), in which healthcare providers and patients together craft a view on the data (Mentis et al., 2017) through collaborative reflection (Schroeder et al., 2017; Figueiredo et al., 2020). Yet, there is also an important difference. In the medical domain, the objectivity of data is typically an asset, whereas, in health coaching, we see that that, particularly, the subjectivity of data is an asset. Health coaches use the data to understand the clients' experience rather than their behavior. Clients and coaches show large efforts to put the data in the right perspective and align their views on the problem and the data. While this happens in medical contexts too, this is amplified in health coaching, as there is less common ground on what the problem is that they are working on, and also less common ground on what the potential benefit of data is in this regard. This makes the process of alignment more prominent and more challenging, compared to using the data “simply” for unraveling disease-related aspects.

### Implications for E-Coaching

It is interesting to consider the implications of our results for e-coaching applications, for example, based on artificial intelligence principles. While our findings highlight the value of a coach-client conversation on the data, not everyone may have access to a human coach. Thus, when designing stand-alone e-coaching, we may try to implement some of these beneficial elements of a conversation with a human coach in other ways.

Across our coaching sessions, data were mostly used as a tool to explore. Specifically, data facilitated talking about, and thus thinking about, what goals a client would have, what wellbeing would mean to her, and possibilities to achieve her goals that would fit her daily life. It is interesting to consider whether a fully automated e-coach could potentially also trigger such a process for a client by herself. Our *data-only* condition, where coaches assessed the client's data in absence of the client, reveals the coaches' unmet information needs that represent the gap that needs to be bridged between the data and appropriate coaching advice. Specifically, the coaches were seeking to understand, among other things, how the client's health data would connect to her goals, the particular challenges she would face while trying to achieve her goals, and the social context of subjective experience of certain activities. While clients can draw from their lived experiences associated with the data, it would still be helpful when e-coaching technology would support clients themselves to reflect on data in deeper and more meaningful ways. A study by Choe et al. (2017) shows that people tend to reflect on their self-tracking data on lower levels, for example, descriptive reflection, and that higher levels of reflection are rarer, for example, transformative and critical reflection (c.f., Fleck and Fitzpatrick, 2010). They argue that these higher levels of reflection are not easy to foster through visual data exploration tools (Choe et al., 2017), while this maybe exactly what is needed to make health data effective in terms of coaching. Kocielnik et al. (2018) offer an interesting and practical solution path to this problem. They designed an application that prompts users, through a conversational agent, to reflect on their data, for example, by asking what happened during peeks or low points in the data, or by asking about goals, motivations, or contexts (Kocielnik et al., 2018). Such use of reflective prompts is promising, given the results of our study, specifically because it leaves the interpretation up to the client, it acknowledges that goals are dynamic, and it avoids value judgments based on data.

### The Use and Expectations of Data Will Be Evolving

We observed that the role of data, and coaches' and clients' expectations of each other and the data, was not yet settled in the coaching sessions. For most coaches and clients, it was the first time they used data in such a way. This is a limitation of our study design, as the coaches' unfamiliarity with the interface and the wearables may have amplified their concerns on the usefulness of the data and inhibited effective use of the data. Still, our study setup represents a realistic scenario, where a client buys a tracker, uses it for a while, visits a coach, and brings her data. So, while, by design of the study, our focus was mostly on the early phases of data sharing, it still gives valid insights into what happens as soon as data are introduced to a coaching process.

It is interesting, though, to consider how this “configuration” of a coach, a client, and data, including their roles and expectations, will possibly evolve over time. Our results show that coaches and clients were largely attentive to how *the others* related to the data. They were interested in what the data would mean to them and tried to understand the others' intentions and expectations on how to use the data in the coaching session. It is likely that coaches' and clients' common ground on these aspects will grow over time when data have been used throughout several coaching sessions. Furthermore, coaches' and clients' data literacy may grow by having more experience in handling and interpreting the data, and this is likely to increase their self-efficacy and feeling of control. When coaches learn about the possibilities and limitations of data, and experience that clients still care about their opinions on top of what the data are representing, this might make coaches more comfortable and willing to use data. As a result, our observations regarding coaches' tendency to refer to health standards or to give the clients a leading role when exploring the data may decrease over time when coaches acquire strategies to effectively utilize the data themselves.

Additionally, Watzlawick et al. (1967) argue drawing from their experience with couples psychotherapy, that the “healthier” a relationship, the more the relationship aspects of communication move to the background, allowing a more dominant role for the subject matter itself. In contrast, with malfunctioning relationships, there is hardly any room for the content, as people are constantly struggling about the nature of the relationship. So, it is expected that, when a good coach-client relationship is maintained and secured, more room is available for discussing the information itself that the data comprise. Indeed, our results show that discussing specific trends in data or drawing actionable insights was not always relevant or appropriate, yet this can shift later in the coaching process. Future research is needed to validate these effects.

## Conclusion

We report on a workshop and field study where we analyzed a variety of health-coaching sessions enriched with a client's self-tracked health data. Observing the role and value of data within a realistic setting enabled us to situate our findings within a broader perspective, including the dynamics of a coaching session. Our results highlight the importance of considering the coach, the client and the data as a whole when evaluating the value of personal tracking data for coaching, or when designing tools that may support this process. Self-trackers and e-coaching applications are not independent computational systems, yet they are embedded in a broader context of health coaching. This constitutes a process where coaches and clients are constantly involved in negotiating interpretations and aligning expectations as they collaboratively work toward health goals. Within this context, self-tracking devices should not be presented as solutions, rather, as helpful tools to support this process.

## Data Availability Statement

The raw data supporting the conclusions of this article will be made available by the authors, without undue reservation.

## Ethics Statement

The studies involving human participants were reviewed and approved by Internal ethical review board of the Human-Technology Interaction group at TU/e Eindhoven. The patients/participants provided their written informed consent to participate in this study.

## Author Contributions

HR and MW conceived the project and supervised data gathering. HR performed primary data analyses. MF acted as a second coder on part of the data. HR wrote the draft manuscript, with several discussions and iterative cycles of improvement on the manuscript by MR, MF, MW, and WI. All authors contributed to the article and approved the submitted version.

## Conflict of Interest

The authors declare that the research was conducted in the absence of any commercial or financial relationships that could be construed as a potential conflict of interest.

## Publisher's Note

All claims expressed in this article are solely those of the authors and do not necessarily represent those of their affiliated organizations, or those of the publisher, the editors and the reviewers. Any product that may be evaluated in this article, or claim that may be made by its manufacturer, is not guaranteed or endorsed by the publisher.

## Footnotes

^1^It was not feasible to apply a multilevel model here, because the question halfway the session (i.e., whether they agreed with the statement “I have enough information to give the client appropriate advice”) was substantially different from the question at the end of the session (i.e., whether they agreed with the statement “Because of the data/the conversation with the client, I have more information than before”). Therefore, we applied a *t*-test to compare the group scores across the information sources data and conversation.
