# Supplementary material for: The Influence of Personal Health Data on the Health Coaching Process
Source: Front Big Data. 2022 Jun 14;5:678061. doi: 10.3389/fdata.2022.678061 (PMC9237329; doi:10.3389/fdata.2022.678061)
Supplement: Supplementary file 1 [file Data_Sheet_1.docx]

Supplementary Material

# Coach-Questionnaire

Coaches filled in this questionnaire twice, first halfway through the session, then at the end. Depending on the condition (*data-first* or *conversation-first*), these questionnaires were targeting their evaluations of data or conversation. We kept the questions as consistent as possible across the different sources of information (data or conversation) and timing (halfway and at the end), to allow for fair a comparison.

[Advice] *Halfway:* What would you advice the client, and why?

*End:* Do you have any additions or changes to your advice? If yes, what would you add or change, and why?

[Confident] How confident are you that this advice will lead to a better result for the client? (5-point scale, ranging from “not confident” to “confident”)

The information resulting from the {conversation / data} is:

[Usable]* 5-point scale, ranging from “not usable” to “usable”.

[Objective] 5-point scale, ranging from “objective” to “subjective”.

[Clear] 5-point scale, ranging from “unclear” to “clear”.

[Relevant]** 5-point scale, ranging from “relevant” to “not relevant”.

[Reliable] 5-point scale, ranging from “unreliable” to “reliable”.

[Enough] *Halfway:* I have enough information to give the client appropriate advice.

*End:* Because of the data / the conversation with the client, I have more information than before. (5-point scale, from “disagree” to “agree”)

[Pers. Exp.] I have a complete picture of the client’s personal experience. (5-point scale)

[Daily Life] I have a complete picture of the client’s behavior in his/her daily life. (5-point scale)

[Supports] {The use of data / Having a conversation with the client} supports my effectiveness as a coach. (5-point scale)

[Value]** What was the value of {the use of data / the conversation with the client} for you as a coach? (Open-ended question)

*Do to new insights, we made small updates on the questionnaire after the workshop, i.e.:*

** In the workshop, we stated “useful” rather than “usable”.*

*** [Relevant] and [Value] was only measured in the field study, not in the workshop.*

# Client-questionnaire

The client-questionnaire was only given to the clients in the field study, not at the workshop.

[Daily Life] I feel like {my coach has / the data represents} a good, complete picture of my daily life and behavior. (5-point scale, from “disagree” to “agree”)

[Pers. Exp.] I feel like {my coach has / the data represents} a good, complete picture of my personal experience. (5-point scale)

[Insight]* I feel like my coach has good insight in me as a person. (5-point scale)

[Understood]* I feel understood by my coach. (5-point scale)

[Value] What was the value of {the conversation with your coach / sharing data} for you? (Open-ended question)

** Only asked after conversation.*
